# Focusing on the mechanism of glycinin-soybean lipophilic protein hybrid gels: Effect of ultrasonic, subunit interactions, and formation process analysis

**DOI:** 10.1016/j.ultsonch.2025.107239

**Published:** 2025-01-19

**Authors:** Yuyang Huang, Jiyuan Liu, Yongping Li, Yongsheng Zhu, Gang Chen, Baoning Zheng, Yixin Zhang, Yang Li, Xiuqing Zhu

**Affiliations:** a Department of Food Engineering, Heilongjiang Key Laboratory of Food Science and Engineering, Heilongjiang Key Laboratory of Grain Food and Comprehensive Processing, Harbin University of Commerce, Harbin 150028, Heilongjiang, China; b College of Food Science, Northeast Agricultural University, Harbin, Heilongjiang 150030, China; c HEILONGJIANG JOYUNG SOYMILK CO.LTD, China; d School of Grain Engineering, Heilongjiang Communications Polytechnic, Harbin 150025, China

**Keywords:** Soybean lipophilic protein, Glycinin subunits, Ultrasonic, Structure, Gel properties

## Abstract

Heat facilitates aggregation and gel formation of soybean proteins. Ultrasonic reduces the size of protein aggregates. This study examined the impact of glycinin (11S) subunits on soybean lipophilic proteins (SLPs) gel formation and underlying mechanisms. Effects of protein dispersion pretreatment with 400 W ultrasonic and associated mechanisms were assessed. Addition of the A- and B-subunits before and after ultrasonic minimally affected SLP secondary structure. A-subunit addition before ultrasonic negligibly affected SLP tertiary structure. Addition of the B-subunit after ultrasonic reduced hydrophobic thermal aggregation. However, the small B-subunit size was unfavorable for the formation of a gel matrix, which led to poor gel properties. In contrast, solubility of the A-subunit after ultrasonic was increased to 31.06 ± 1.62 %). Particle size was decreased to 43.33 ± 1.36 nm for A:SLP (1:2). Endogenous fluorescence spectroscopy demonstrated increased protein unfolding after ultrasonic and decreased disulfide bonds. These changes improved the gel state. Rheological and microstructural analyses revealed increased energy storage modulus and yield strain, accompanied by a more homogeneous microstructure following ultrasonic. Microscopic improvement resulted in increased encapsulated water within interstitial spaces of the A-SLP gel matrix. This enhanced water mobility in B-SLP gels, in turn weakening gel stability. The changes observed in B-SLP were primarily due to reduced hydrophobic interactions between the proteins. The findings clarify the effect of ultrasonic treatment on the formation of soybean globulin-SLP hybrid gels at the subunit level. The data provide a theoretical basis for the synergistic utilization of soybean proteins among different components.

## Graphical abstract

**Figure:**
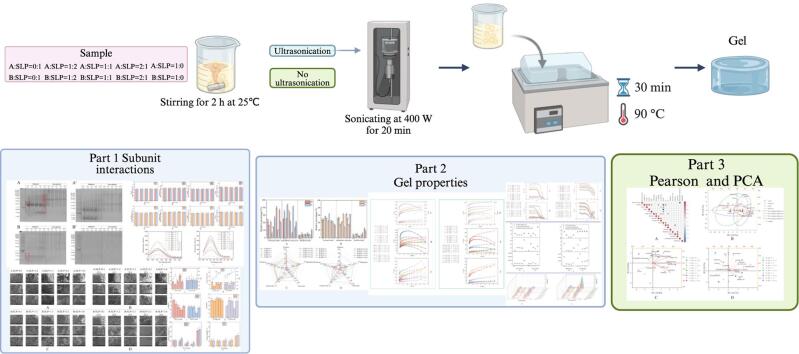

## 1 Introduction

Soybean lipophilic proteins (SLPs), which comprise 30 % of soybean isolates, are lipoprotein complexes composed of oil and a variety of proteins containing a large number of phospholipids. SLPs are mainly basic and hydrophobic proteins with relatively low molecular weights (24, 18, and 17 kDa). They also contain soybean globulin (11S) and β-conglycinin (7S) [1], [2], [3], [4], which also confer certain gelling properties on SLPs. The 11S globulin (300–380 kDa), which comprises approximately 30 % of soy protein isolate (SPI), is a hexameric protein consisting of an A-subunit (∼35 kDa) linked by a disulfide bond to a specific B-subunit (∼20 kDa) and a set of AB-subunits that form a trimer by hydrophobic and/or hydrogen bonding. The two trimers are then superimposed, resulting in the formation of a soybean globulin hexamer by hydrogen bonding and electrostatic interactions [5], [6]. Several studies have focused on gel properties, such as pH, salt, and heat-induced 11S, 7S, and 11S/7S ratios [7], [8], [9]. The 7S fraction contains a greater proportion of non-network proteins and exhibits a slower gelation rate, predominantly by forming flocs that reinforce the gel backbone. Consequently, the gel displays enhanced elasticity when the 7S content increases [9], [10]. The heat treatment of soy proteins with a high proportion of 11S results in the formation of a significant number of aggregates with larger particle sizes. Furthermore, the number and thickness of chains within the gel network increase with the proportion of 11S globulin, indicating a pivotal role of 11S in the development of large aggregates [11]. The accelerated gelation rate of 11S results in the formation of larger aggregates and higher gel strength [12]. Wu et al. [9] also showed that the high hydrophobicity of the B-subunit in 11S globular proteins plays a significant role in the development of aggregates of large particles.

The process of gel formation by 11S can be summarized as follows. The B-subunit first undergoes thermal aggregation driven by hydrophobic interactions and forms a gel matrix. The A-subunit forms thermal aggregates and complements the gel matrix through disulfide bonding. Hydrogen bonds form during cooling, resulting in the formation of a gel structure [13]. The gel formation process of soy protein has become a topic of considerable interest within the research community, with a particular focus on the role of its subunits. However, characterization of SLP gel properties has not been extensively studied [7]. Sirison et al. [13] observed that SLP emulsions exhibited a reversible liquid–solid transition upon acidification within the weak acid pH range of 7.2–4.1, and an emulsion gel state within the pH range of 5.6 to 4.5. These findings suggest that adjustment of pH is a prerequisite for the formation of an SLP gel [14]. Zhong et al. [7] comprehensively described the manufacturing process, physical characteristics of the gel, and investigated the release of nutrients during the in vitro digestion of a thermosensitive emulsion gel composed of SLP-hydroxypropyl methylcellulose (HPMC) and calcium chloride. Analysis of the infrared, X-ray diffraction, and 1H nuclear magnetic resonance (NMR) spectra demonstrated that the introduction of CaCl_2_ resulted in alterations to the crystalline structure of the SLP-HPMC composite [15]. Furthermore, a substantial body of evidence indicates the importance of examining the gelling properties of SLPs and the impact of the ratio of 11S, 7S, and SLPs on the gelling properties of soy proteins [16].

The use of physical methods to modify the structure and enhance the functional properties of proteins is a common approach to optimize the texture and taste of food products. Ultrasonic is a food processing technique with minimal environmental impact. Moderate-amplitude, short-duration ultrasonic modification is more effective and has a favorable effect on the gel properties of SPI [17]. As a non-thermal processing method, ultrasonic can be used to process liquids, dispersions, and solids by varying the frequency and intensity to promote optimal timing as well as enhance energy and mass transfer, resulting in improved food yield. Cavitation and high shear energy waves generated by ultrasonic caused deformation and partial denaturation of the protein molecules, resulting in exposure of surface active sites, increased free sulfhydryl content and surface hydrophobicity, thus enhancing the subsequent thermally induced aggregation and gelation behaviour of soy protein [18]. It was also found that combined homogenisation and ultrasonic treatments disrupted the insoluble precipitate formed by the B-subunit of 11S and increased the solubility of SPI, further enhancing the thermally induced gelation of SPI [19]. The utilization of high-intensity ultrasonic as a pretreatment, based on the exemplary processing characteristics of soy proteins, represents an alternative method for enhancing the gelling properties of soy proteins, achieving superior outcomes in terms of optimized texture and mechanical properties and more compact gels with enhanced hydrodynamic properties. This method can be employed in the food industry [20].

The gel formation from 11S results in a rough texture and larger pores when heated [21]. In contrast, the high lipid content of SLPs, particularly phospholipids, results in low protein content and poor solubility upon heating. This presumably leads to poorer gel formation by SLPs [22]. In summary, 11S tends to form aggregates with large particle size and poor solubility of SLP, and the cavitation and shear effects of ultrasonic were used to prevent protein aggregation and improve solubility. At the same time, modification of SLPs and 11S subunits using ultrasonic remains an understudied area, and existing research on the gelation mechanism in an ultrasonic environment is not yet comprehensive. In this paper, different ratios of soy protein gels were prepared using medium power (400 W) ultrasonic pretreatment and the effect of ultrasonic treatment on the formation of soy globulin-SLP hybrid gels at the subunit level was elucidated, highlighting the importance of ultrasonic effects in the synergistic exploitation of soy proteins between different components. Thus, the comprehensible conclusions of this study provide valuable insights into the possible use of ultrasonic treatment of soy proteins to modulate their physical and chemical structures.

## 2 Materials and methods

### 2.1 Materials

Cold-pressed soybean meal was purchased from YUWANG Group Co., Ltd. (Yucheng, Shandong, China). Tris-base and β-mercaptoethanol were purchased from Beijing Solarbio Science & Technology Co., Ltd. (Beijing, China). Analytical grade chemicals and reagents, including hydrochloric acid (HCl, 36 %), sodium hydroxide (NaOH, ≥99 %), sodium phosphate monohydrate (NaH_2_PO_4_·2H_2_O, ≥99 %), sodium phosphate dihydrate (Na_2_HPO_4_·2H_2_O, ≥98. 5 %), sodium chloride (NaCl), sodium dodecyl sulfate (SDS, ≥98.5 %) and urea (≥99.5 %) were purchased from Sinopharm Chemical Reagent Co. Ltd (Beijing, China).

### 2.2 Preparation of A and B subunits, and SLP

#### 2.2.1 Isolation of 11S and SLP proteins

The protein contents of 11S and SLP, determined by the Kjeldahl method, were 92.38 ± 0.86 % and 81.71 ± 0.09 %, respectively. The cold-pressed soybean meal were dispersed with 0.05 mol/L Tris-HCl pH 8.5 buffer in 15-fold dispersions. The mixtures were then stirred at 40 °C for 1 h, and extracted using a TG16-WS centrifuge (Xiangyi Laboratory Instrument Development Co., Ltd.) at 500 g for 20 min to obtain the supernatant and precipitate. First, the pH value of the supernatant was adjusted to 5.8 using a PHS-25 pH meter (Shanghai Yidian Scientific Instrument Co., Ltd.), and the precipitation of 11S was achieved by centrifugation (500 g for 20 min) [23]. Another serving of cold-pressed soybean meal is dry-heat treated in an oven at 70 °C for 2 h. After removing 11S as described above, the supernatant was adjusted to pH 5.0, heated at 55 °C for 15 min, its pH was adjusted to 5.5, and it was centrifuged at 500 g for 20 min. The resulting precipitate was SLP [1]. The protein was extracted using an ALPHA 1–2 LD plus freeze dryer (CHRIST, Germany). The protein fractions were freeze-dried and prepared for use.

#### 2.2.2 Purification of 11S

Agarose gel CL-6B was employed for gel filtration chromatography. CL-6B was loaded gradually and uniformly onto a 4.5 × 25 cm column, which was then equilibrated with 1000 mL of 35 mmol/L phosphate buffer solution (pH 7.6). One gram of lyophilized 11S was dissolved in 6 mL of the sample loading buffer for gel filtration chromatography, which is also referred to as the equilibration buffer. Elution was performed with the buffer solution using a 6-min collection time for each tube. A total of 50 tubes were collected. In accordance with the findings of Iwabuchi et al. [24] the protein exhibiting the most extensive range of distribution and the earliest flow out was the target-purified protein, which was identified as 11S globulin. Subsequently, the corresponding solution was dialyzed with ten times the volume of deionized water. The water was replaced every 8 h three times over a 24-h period. The preparation was freeze-dried and lyophilized. lyophilized samples were sealed and stored.

#### 2.2.3 Isolation of the A and B subunits

11S globulin (5 g) was dissolved in 1000 mL of 30 mM Tris buffer (pH 8.0) containing 15 mmol/L α-mercaptoethanol. The protein solution was heated at 90 °C for 30 min in a water bath, followed by centrifugation at 4000 rpm for 20 min. The precipitate was washed twice with 30 mM Tris buffer (pH 8.0), suspended in distilled water, freeze-dried, and collected as the basic B subunits. The supernatant was freeze-dried and collected as the acidic subunit A.

### 2.3 Preparation of gel samples

According to our experiments (not shown in this paper), 15 % is the concentration of SLP that alone can form a viscoelastic gel. A-SLP and B-SLP dispersions (15 % wt%) were prepared by mixing the A and B subunits with SLP at ratios of 0:1, 1:2, 1:1, 2:1, and 1:0 (w/w), and stirring for 2 h at 25 °C. For another set of sonicated samples, using the same ratio and concentration, the sonicated pretreated samples were sonicated in an ice bath using a model LC-CB-1000E sonicator (LICHEN, Suzhou, China) at 20–25 kHz using a 6 mm titanium-tipped probe for the processing of the A-SLP and B-SLP dispersions. The ultrasonic conditions were based on those described by Zhang et al. [25] with minor modifications. Specifically, the samples were sonicated at 400 W for 20 min with an on–off pulse duration of 2 s on and 2 s off. The sonicated pre-treated samples were designated as A-SLP(U) and B-SLP(U).

Subsequently, the solutions were left overnight at 4 °C to permit the complete hydration of the proteins. All protein solutions were transferred to airtight containers and heated to 90 °C for 30 min in a thermostatic water bath. Samples were immediately cooled in ice-cold water to prevent further denaturation.

### 2.4 SDS polyacrylamide gel electrophoresis (SDS-PAGE)

The loading buffer for reduction electrophoresis consisted of 50 mL water, 20 mL 10 % SDS solution, 12.5 mL 0.5 M Tris-HCl buffer (pH 6.8), 5 mL β-mercaptoethanol, 10 mL glycerol, and 2.5 mL 2 % bromophenol blue. For non-reducing electrophoresis, the β-mercaptoethanol was replaced with deionized water. The staining solution consisted of 1 g Coomassie brilliant blue, 450 mL methanol, and 100 mL glacial acetic acid diluted with distilled water to 1 L. The decolorization solution consisted of 454 mL methanol, 74 mL glacial acetic acid, and 472 mL distilled water. The concentrations of the separation and concentration gels were 12 % and 5 %, respectively. The protein solution (3 mg/mL) was diluted 1:1 with sampling buffer after allowing the reaction to proceed for 4 h, and the samples were boiled for 10 min. All soy protein samples (10 μL) were loaded onto the concentration gel in an electrophoresis apparatus for protein separation.

### 2.5 Fourier-transform infrared spectroscopy (FTIR)

A Spectrum Two infrared spectrometer (PerkinElmer, Waltham, MA, USA) was used to analyze protein conformation in soy protein gels mixed with varying ratios of 11S, 7S, and SLP. Samples were freeze-dried, collected in small amounts, and placed on germanium (Ge) crystals in a horizontal attenuated total reflectance instrument. Each sample was flattened, covered with Ge crystals (2 mm in diameter), and scanned for 1 min to obtain IR spectra.

### 2.6 Fluorescence spectroscopy

Endogenous fluorescence spectroscopy of lyophilized protein powder was performed using an F-6000 fluorometer (Hitachi Ltd., Tokyo, Japan). Lyophilized gel samples were diluted to 0.1 mg/mL with distilled water. The fluorescence emission spectra were recorded at an excitation wavelength of 290 nm, emission wavelengths ranging from 300 to 450 nm, scanning rate of 1000 nm/min, and slit widths of 5 nm for excitation and emission [21].

### 2.7 Scanning electron microscopy (SEM)

Samples with a length and width of 5 mm and a thickness of 1 mm were collected, dehydrated, fixed for 24 h, and freeze-dried using an Alpha 1–4/ldplus device (Martin Christ Gefriertrocknungsanlagen GmbH). The freeze-dried samples were sputtered with 10 nm gold/palladium and examined by SEM using a model s-3400n microscope (Hitachi).

### 2.8 Particle size and zeta (ζ)-potential

Protein solutions of 1 mg/mL were prepared and measured at 25 °C with a Zetasizer Nano laser particle size analyzer (Malvern Instruments, Malvern, UK), using a 5 mmol/L phosphate buffer solution (pH 7.0).

### 2.9 Protein solubility

The buffer systems used for dissolving proteins included 0.035 mol/L phosphate buffer solution (pH 7.6) and phosphate buffer containing SDS (1.5 g/100 mL), urea (8 mol/L), or β-mercaptoethanol (0.1 mol/L). SDS, urea, and β-mercaptoethanol disrupted intermolecular hydrophobic interactions, hydrogen bonds, and disulfide bonds, respectively. Each specific chemical bond and its interactions were analyzed by simple mathematical operations based on the above protein solubility analysis [26]. Hydrophobic interactions: (2)–(1), hydrogen bonds: (3)–(1), disulfide bonds: (4)–(1). Numbers in parentheses indicate the solubility of the proteins in the corresponding solution.

Each sample (0.05 g) was placed in a centrifuge tube, 4 mL of extraction solution was added, and the mixture was shaken for 2 h. The mixture was centrifuged at 4000 rpm for 10 min, and 20 μL of supernatant were collected, mixed with 180 μL of deionized water, and 10 mL of G250 were added to the centrifuge tube. After stabilization for 2 min, the soluble proteins in the supernatant were analyzed using the Lowery method at 595 nm using an Alpha-1506 UV–Vis Spectrophotometer (Shanghai Spectrum Instrument Co., Shanghai, China). Protein solubility was calculated as the ratio of soluble protein in the supernatant to the total protein content of the sample.

### 2.10 Determination of texture properties

A New Plus texture instrument (ISENSO, Torrence, CA, USA) was used for texture profile analysis. Pre-testing, testing, and post-testing speeds were all 1 mm/s. The test time was 5.0 s. Gel samples 10 mm in diameter and 10 mm in height were compressed with a trigger force of 5.0 g at 30 % of the target value. Gel strength (N/m^2^) was defined as the maximum force for sustained compression.

### 2.11 Determination of rheological properties

Rheological measurements were performed using an MCR 102 Advanced Rotational Rheometer (Anton Paar Trading Co., Ltd., Graz, Austria) equipped with 25 mm parallel plates. The gap between the parallel and base plates was adjusted to 1 mm. Excess sample was carefully removed and the edges of the gel sample were covered with silicone oil to prevent water evaporation. Rheological measurements were performed after sample loading. The energy storage modulus (G′) and loss modulus (G′′) were recorded in the frequency range of 0.1 to 10 Hz with a linear viscoelastic (LVE) strain of 0.5 %. The trends of G′ and loss angle with frequency were calculated. Large amplitude oscillatory shear testing was performed using strain sweeps ranging from 0.01 to 1000 % at 1 Hz. The following equation was used to calculate the cohesive energy density (E_c_) [27]:

(1) $$\begin{array}{c} E_{c}=\frac{1}{2}{\gamma_{c}}^{2}G_{cr} \end{array}$$

where γ_c_ is the critical strain and G_cr_ is the storage modulus at the critical strain.

### 2.12 Low-field NMR

Spin relaxation time (T2) was measured using an NMI20 MRI analyzer (Neumay Electronic Technology Co., Ltd., Shanghai, China). Approximately 2 g of the gel sample was placed in a 25 mm cylindrical glass tube. The value of the pulse interval τ (time between 90° and 180° pulses) was 150 μs. The number of collected echoes and scans were set to 3000 and eight, respectively. All relaxation measurements were performed at 25 °C.

### 2.13 Statistical analysis

All experiments were repeated three times. Data are expressed as mean ± standard deviation. Data were statistically analyzed using one-way analysis of variance and significance was analyzed using Tukey's test (p < 0.05). Origin 2019 software (OriginLab, Northampton, MA, USA) was used to plot data.

## 3 Results and discussion

### 3.1 Physicochemical properties

#### 3.1.1 Protein electrophoresis analysis

**Fig. 1:**
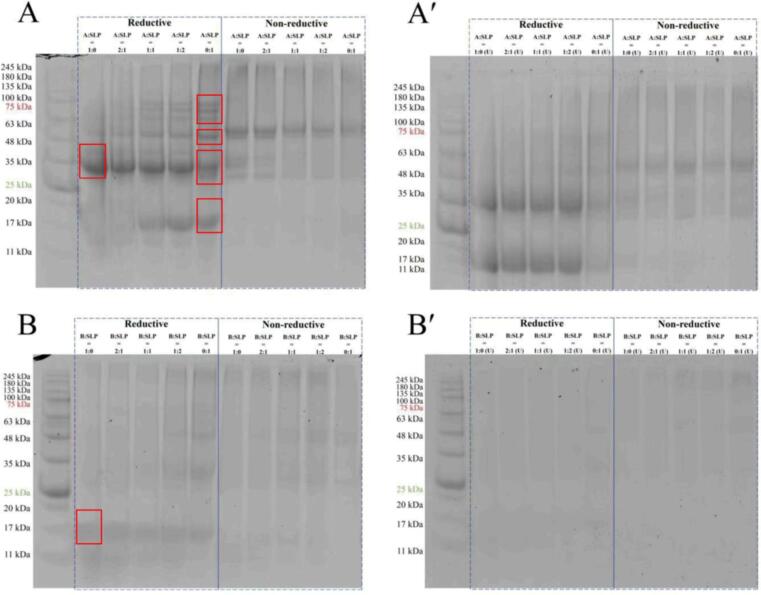
Non-reductive and reductive SDS-PAGE profiles of soy protein gels before and after ultrasound.(Figure A shows the reductive and non-reductive electrophoresis of different proportions of A-SLP before sonication and Figure A' shows the reductive and non-reductive electrophoresis of different proportions of A-SLP after sonication; Figure B shows the reductive and non-reductive electrophoresis of different proportions of B-SLP before sonication and Figure B' shows the reductive and non-reductive electrophoresis of different proportions of B-SLP after sonication).

The reducing agent (β-mercaptoethanol) disrupts the disulfide bonds between proteins, facilitating the dissolution of aggregates that form the gel. Consequently, comparison of the pattern produced by reducing and non-reducing gel electrophoresis can reflect the molecular weight distribution of aggregates connected by disulfide bonds. Under reducing conditions, the isolated acidic A subunit displayed two predominant bands with molecular weights of approximately 42 kDa (A3) and 36–38 kDa (A1a, A1b, A2, and A4) (Fig. 1A, Reductive A:SLP = 1:0) [28]. In contrast, the basic B subunit displayed a distinctive band with a molecular weight of approximately 15–20 kDa [29] (Fig. 1B, Reductive B:SLP = 1:0). Furthermore, SLP, which is primarily constituted of globin (11S) and β-companion globin (7S), exhibited four prominent bands of 63–75 kDa (αα′-subunits), 48 kDa (β-subunits), 25–35 kDa (A-subunits), and 15–20 kDa (B-subunits) (Fig. 1A, Reductive A:SLP = 0:1).

From the data presented in Fig. 1, it can be concluded that the distribution of aggregates connected by disulfide bonds in A-SLP gels was concentrated, whereas the molecular weight bands of aggregates connected by disulfide bonds in B-SLP gels were more dispersed. Additionally, aggregates smaller than 11 kDa were observed in the B-SLP gels, which is not conducive to the formation of a continuous and homogeneous gel network structure for low-molecular weight proteins. Secondly, a comparison of reduction electrophoresis before and after ultrasonic revealed that strong mechanical ultrasonic cavitation resulted in the decomposition of the A-subunit, B-subunit, and SLP into smaller units [30]. This resulted in a more concentrated distribution of the protein molecular weights in the A-SLP gel following ultrasonic. Furthermore, B-SLP retained a minor proportion of its initial molecular weight bands following ultrasonic, which was ascribed to the more pronounced decomposition of the B-subunit. Additionally, the gel contained minor units < 11 kDa. A comparison of non-reducing electrophoresis before and after ultrasonic revealed that the molecular weight distribution of the gel remained largely unchanged following ultrasonic treatment. This finding is supported by previous observations [29]; the authors noted that ultrasonic-induced physical modifications at the macromolecular level did not significantly alter the molecular weight distribution. The findings demonstrate that non-covalent interactions, including electrostatic and hydrophobic forces, are the primary driving forces behind protein aggregation in soy protein hydrolysates following ultrasonic treatment.

In general, the incorporation of the A and B subunits into SLP led to a more concentrated distribution of molecular weight bands and weakening of the larger and smaller molecular weight bands. It is mean that the production of the proteins was less, so the bands were less prominent on the gels. Ultrasonic resulted in a more concentrated distribution of the two molecular bands of A-SLP, but did not reduce their molecular weight. Conversely, ultrasonic resulted in gradual hydrolysis of the low molecular weight peptide fragments of B-SLP to smaller molecular weights.

#### 3.1.2 Secondary structure

**Fig. 2:**
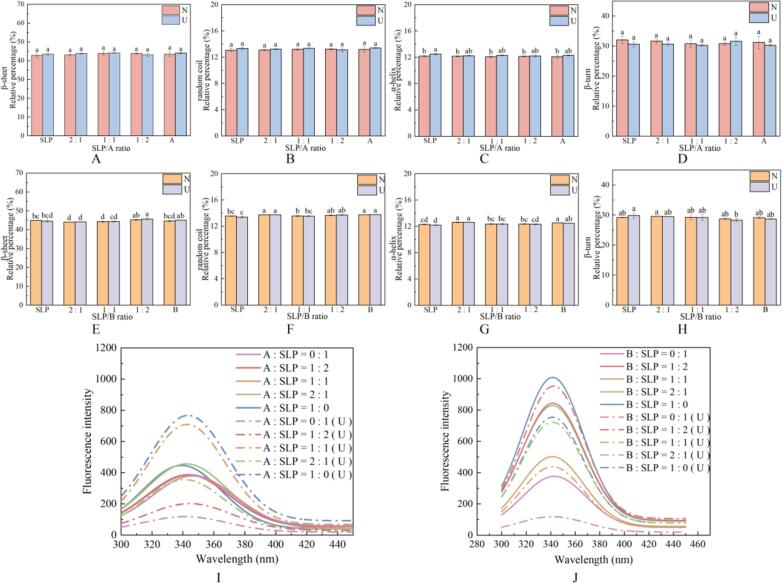
Secondary structure (A-H) and intrinsic fluorescence emission spectra (I-J) of mixed gel system with different ratios of A, B, and SLP.

Alterations in the secondary structure of the proteins present in the A- and B-SLP gels were determined by deconvolution and curve fitting of the amide I region. The primary structure of the composite protein gels was identified as the β-sheet structure, indicating that ultrasonic had an impact on the secondary structure of A-SLP and B-SLP (Fig. 2A-D, E-H).

The β-sheet content of the proteins in the A-SLP gels was higher both before and after ultrasonic. The β-sheet structure is the primary structural component that maintains the A-SLP system, and the gels form a more ordered and stable structure [29], [31]. The application of ultrasonic disrupted the β-sheet structure of the A:SLP = 2:1 gel, leading to the conversion of this structure to a β-turn and the destruction of the ordered and stable structure. In contrast, at other ratios, the content of β-turn was reduced, while the content of β-sheet was increased.

The addition of the B-subunit to SLP increased contents of α-helix and random coil before and after ultrasonic. The findings suggest that the interaction of the B-subunit with SLP enhances the rigidity of the structure and increases the number of disordered regions [32]. However, the highest content of β-sheet of the four structures was observed at the B:SLP ratio of 2:1 (45.26 ± 0.17 % before ultrasonic and 45.6 ± 0.45 % after ultrasonic), which enhanced the order and stability of the gel.

Summarizing the findings, the addition of the A-subunit to SLP increased in the α-helix content and a corresponding increase in gel rigidity. Conversely, the addition of the B-subunit increased β-sheet content and decreased β-turn content, resulting in a more ordered and stable structure. The application of ultrasonic had no discernible effect on the A-SLP gels. However, ultrasonic increased the β-sheet structure and notably enhanced the orderliness of the B-SLP.

#### 3.1.3 Tertiary structure

Fluorescence spectra can reflect alterations in the polarity of the microenvironment of hydrophobic amino acids, which in turn affect the tertiary structure. The maximum fluorescence emission occurred between 320 and 350 nm, indicating that tryptophan was dominant. The fluorescence intensity of B-SLP was greater than that of A-SLP before ultrasonic, indicating a higher degree of protein unfolding in B-SLP gels (Fig. 2I, J). This finding aligns with the observations made for the B-subunit in a previous study [21]. Notably, the difference in the fluorescence spectra among A-SLP samples was minimal, with relatively limited influence of the A −subunits on the degree of SLP unfolding.

Following ultrasonic, the fluorescence peaks exhibited an upward shift at A:SLP ratios of 1:1 and 1:0, and at a B:SLP ratio of 1:2. This may be because the hydrophobic amino acid residues were in a less polar environment due to polymerization, aggregation, or peptide-peptide binding, which facilitated the unfolding of the protein structure. For example, exposure to hydrophobic amino acids enhances the interactions between residues, resulting in the formation of gels that are prone to the formation of larger, insoluble aggregates due to hydrophobic interactions [33], [34]. Conversely, the fluorescence peaks shifted downward at other ratios, indicating that the polarity of the environment around the hydrophobic amino acid residues increased. Furthermore, a redshift in the λmax following ultrasonic was observed in gels comprised of SLP and A-subunit at a ratio of 1:2, and in gels with the same ratio of B to SLP. These findings suggest a more loosely packed tertiary structure and provides evidence of a greater degree of unfolding of the protein structure [17].

The collective findings indicate that the incorporation of the B-subunit markedly enhanced protein unfolding within the gel matrix compared to the A-subunit. This was accompanied by a reduction in protein aggregation, predominantly driven by hydrophobic interactions. Ultrasonic facilitated the formation of a more hydrated protein structure.

#### 3.1.4 Morphology of soy protein gels

**Fig. 3:**
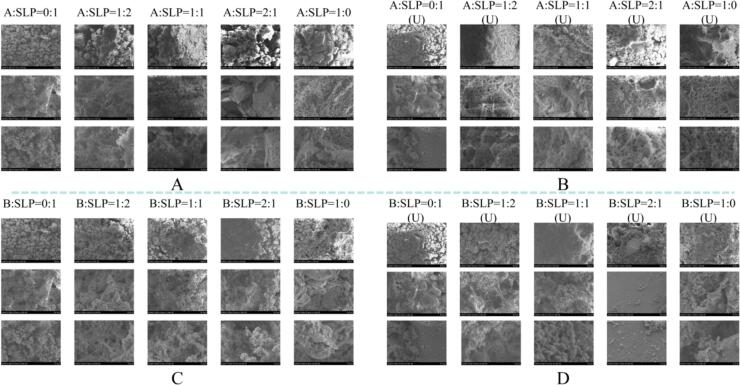
Morphology of gels with different ratios of A, B, and SLP. (Fig. A is the A-SLP gel before sonication, Fig. B is the A-SLP gel after sonication, Fig. C is the B-SLP gel before sonication, and Fig. D is the B-SLP gel after sonication).

SEM enabled the direct observation of the three-dimensional network gel structures formed in different samples after ultrasonic (Fig. 3). In general, the structure of the gel network is related to chain thickness and therefore to protein aggregate size [35]. Before ultrasonic, the addition of A-subunits increased in the particle size within the gel. The addition of B-subunits resulted in decreased particle size. The findings are consistent with the particle size results. As the contents of the A and B subunits increased, the reticular network structure within the gel also increased, particularly in the case of A:SLP = 1:1 and B:SLP = 1:1. The addition of the A-subunit enhanced the gel network thickness, whereas the addition of the B-subunit resulted in a maintenance of the gel network thickness.

Secondly, it was evident that the pores of the gels before ultrasonic were larger, exhibiting rough and uneven surfaces. In contrast, the pores in sonicated gels were significantly smaller. Gels formed from A-SLP exhibited notable surface granularity and substantial pore size before ultrasonic. Subsequent to ultrasonic, the gel network manifested a non-compact, flocculated structure, a phenomenon that was particularly pronounced in gels with an A:SLP ratio of 2:1. With the addition of the B-subunit, when B:SLP = 1:1, the gel network after ultrasonic became ordered and homogeneous, with similarly sized cavities uniformly distributed in the network, corresponding to the results of texture profile and rheological properties, and its gel quality was improved. Ultrasonic facilitates the unfolding of protein structures and the interaction of protein molecules with water, which is conducive to the formation of smaller pore structures [17].

#### 3.1.5 Average size and polydispersity index (PDI)

To gain further insight into the impact of varying conditions on the proteins within the gels, the mean particle size and PDI of the A- and B-SLP protein aggregates were determined using a particle size analyzer. The average particle size of the protein aggregates demonstrated a tendency increased and then decreased, with larger and smaller particle sizes observed for A-SLP and B-SLP gels, respectively. This aligns with the molecular weight distributions observed in non-reduced electrophoresis. A smaller particle size of the B-subunit is conducive to enhanced gel elasticity, while a particle size that is too small may impede gel formation.

Following ultrasonic, the average particle size of the aggregates increased, accompanied by a more concentrated particle size distribution. A previous study [25] observed that in preparations of proteins that had been broken by ultrasonic the enhancement of water–water interactions resulted in the close proximity and aggregation of the protein fragments. In the present study, when the A:SLP ratio was 2:1, the average particle size of the aggregates decreased after ultrasonic. The reductions in particle size and PDI are conducive to the formation of a compact and homogeneous gel network, which is beneficial for the stable retention of water within the gel [36]. Therefore, when preparing sonicated protein gels, ultrasonic promotes protein aggregation, thermal aggregation, and swelling, leading to an increase in aggregate particle size [37], [38], as was presently observed in gels with A:SLP = 1:2, B:SLP = 1:2, and B:SLP = 2:1, and B-subunit gels. Notably, the difference in the average particle size of the samples within the B-SLP group after Ultrasonic was relatively insignificant, indicating that the gel properties of the gels formed after ultrasonic pre-treatment were not significantly influenced by the particle size.

The maximum particle size was observed when the A-subunit was added to SLP at a 2:1 ratio. However, this larger particle size was not conducive to the intermolecular cross-linking of proteins. Furthermore, the formation of gels with strong rheological and textural properties was not straightforward. Given that the secondary and tertiary structures of A-SLP are not the primary determinants of gel properties, it can be postulated that alterations in particle size may be the principal factor influencing the gel properties of SLP as a result of the addition of the A-subunit. While there was no significant change in the average particle size after the addition of the B-subunit to SLP, and the significance before and after ultrasonic was only marginal, the particle size was reduced, resulting in a decrease in gel strength. This is because large and compact protein aggregates act as precursors for gel formation, whereas smaller aggregates are detrimental to gel matrix [39].

#### 3.1.6 ζ-potential

**Fig. 4:**
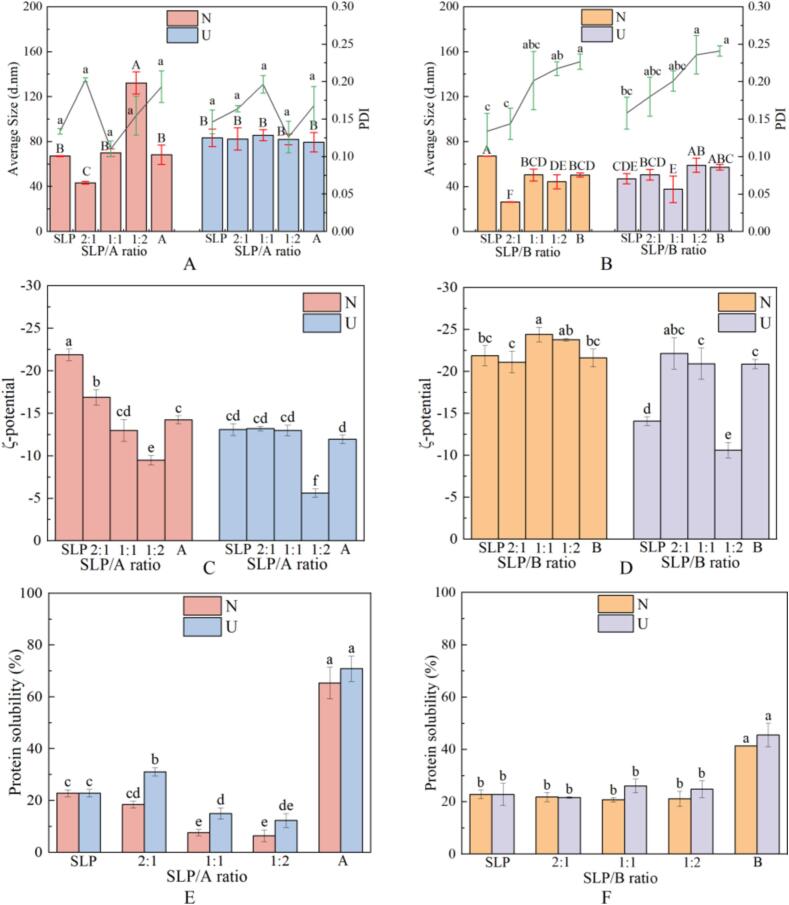
Average size and PDI (A, B), ζ-potential values (C, D), and solubility (E, F) of mixed gel with different ratios of A, B, and SLP.

The ζ-potentials of all gel samples were negative (Fig. 4C, D), indicating that there were more negatively charged amino acids than positively charged amino acids on the surface of the proteins [40]. The absolute values of the potentials of the mixed system of A and SLP were reduced by ultrasonic, likely due to the binding of A to SLP at the charged sites. This would result in a lower proportion of charged groups on the surface of the proteins, leading to a reduction in the electrostatic repulsion between proteins. This would promote the aggregation of proteins, in turn leading to the formation of more stable aggregates and cross-linked structures.

Similarly, the absolute value of the ζ-potential of the aggregates in B-SLP gels was larger when the ratio of B:SLP was 2:1 before ultrasonic exposure, and decreased to 10.60 ± 0.94 mV following ultrasonic exposure. The cavitation effect of ultrasonic resulted in a significant reduction in the ζ-potential and dissociation of the aggregates due to the mechanical stresses. This led to an increase in particle size. Conversely, when the B:SLP ratio was 1:2, the absolute value of the ζ-potential increased after ultrasonic. This finding suggests that the protonation of protein molecules exposes positively charged amino acids inside the protein. This occurs after incomplete neutralization of the negative charge on the protein surface, thereby reducing electrostatic repulsion or aggregation of the protein. This may result in the destruction of insoluble aggregates and a reduction in particle size. This suggestion is supported by previous studies [34], [39], [41].

#### 3.1.7 Solubility

The protein solubility in the gel exhibited a trend comparable to that of particle size and ζ-potential (Fig. 4E, F). Addition of the A-subunit to SLP resulted in decreased solubility, which was attributed to both electrostatic repulsion and the formation of insoluble aggregates. In contrast, the addition and content of the B-subunit had minimal impact on the solubility of proteins in the gel system. The proteins in the B-SLP gels exhibited relatively high solubility. This can be attributed to two factors: the smaller particle size of the B-subunit and the presence of a minor component of non-removable 7S in SLP. The solubility of the basic B-subunit of 11S is enhanced following interaction with 7S [42], [43].

Ultrasonic can alter protein structure, exposing hydrophilic regions and enhancing water-protein interactions [44]. In the present study, this was particularly evident in A-SLP gels, where the disulfide bond content was reduced after ultrasonic, thus confirming that the conversion between insoluble precipitates and soluble components was associated with the rupture of the disulfide bond. Furthermore, the reduction in A-SLP before and after ultrasonic demonstrated that ultrasonic resulted in a reduction in protein molecular weight, providing additional evidence that ultrasonic altered both covalent and non-covalent interactions within the gel [45].

### 3.2 Gel properties

#### 3.2.1 Different intermolecular forces in soy protein gels

**Fig. 5:**
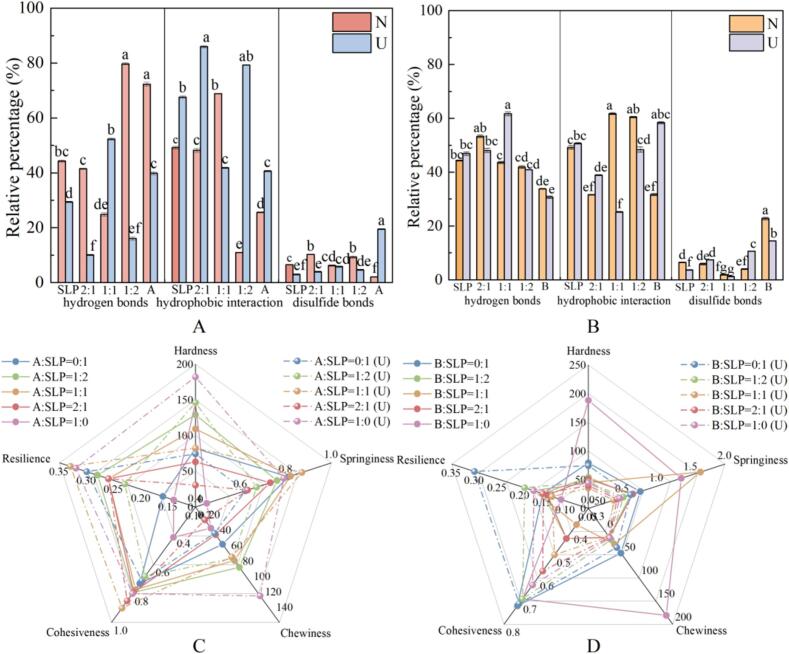
Different intermolecular forces (A, B) and TPA (C, D) of mixed gel with different ratios of A, B, and SLP.

The intermolecular forces were derived from the solubility of the A-SLP and B-SLP gels in different solvents (Fig. 5A, B). In general, hydrogen bonding plays a significant role in the formation of A-SLP gels before ultrasonic, whereas hydrophobic interactions become the dominant forces following ultrasonic. In contrast, both hydrogen bonding and hydrophobic interactions contributed to the formation of B-SLP gels before and after ultrasonic. Furthermore, ultrasonic enhanced the strength of the hydrophobic interactions in B-SLP gels. However, ultrasonic can intensify the denaturation and aggregation of unfolded proteins, which in turn leads to the externalization of hydrophobic sites and an increase in hydrophobic interactions [29].

Before ultrasonic, the formation of the gel network was dominated by hydrophobic interactions when the ratio of A to SLP was 1:1. Conversely, the addition of the A-subunit resulted in the dominance of hydrogen bonding. However, when the A-subunit content was increased (A:SLP = 2), hydrogen bonding played a dominant role in the gel network formation. 1. Following the addition of B-subunit, hydrogen bonding was identified as the dominant mechanism in the formation of gels when B:SLP = 1:2. This was also observed when the content of B-subunit was increased to B:SLP = 1:1, indicating that the A and B subunits exhibited disparate mechanisms in the formation of gels in SLPs. This further indicated that the A and B subunits use different mechanisms for gel formation by SLP, with hydrophobic interactions playing a dominant role.

Ultrasonic disrupted hydrogen bonds and enhanced hydrophobic interactions in A-SLP gels, consistent with the results of hydrophobic thermal aggregation in the tertiary structure. In contrast, the opposite trend was observed for B-SLP gels, particularly for the B:SLP ratio of 1:1. Ultrasonic resulted in a notable increase in the disulfide bond content of the A-subunit gels (A:SLP = 1:0, B:SLP = 1:2, and B:SLP = 2:1). This observation may be attributed to the exposure of free sulfhydryl groups within the protein molecule, facilitated by ultrasonic treatment. The formation of thermal aggregates involves the generation of disulfide bonds between adjacent free sulfhydryl groups via oxidation [46]. It has been demonstrated that increasing the content of intermolecular disulfide bonds and decreasing the free sulfhydryl groups significantly enhances the rheological properties of SPI gels, resulting in denser and more homogeneous three-dimensional gel networks [47], [48].

#### 3.2.2 Texture profile

Textural properties of the gels are shown in Fig. 5C and 5D. These properties include hardness, elasticity, chewiness, cohesion, and repairability. Before ultrasonic, the gels exhibited an increasing trend in all these properties with the incorporation of A-subunit at a content of 81 %. The textural properties of the gels were 98 ± 5.58, 0.81 ± 0.01, 48.41 ± 1.86, 0.73 ± 0.05, and 0.16 ± 0.01, respectively for A:SLP = 1:2 and increased to 129.18 ± 12.44, 0.82 ± 0.052, 78.39 ± 17.54, 0.7921 ± 0.048, and 0.28 ± 0.04, respectively, for A:SLP = 1:1) following ultrasonic. The textural properties of the gels were enhanced following ultrasonic, resulting in maximum values of elasticity, cohesion and restitution of the gels (0.87 ± 0.01, 0.90 ± 0.02, and 0.33 ± 0.02, respectively, for A:SLP = 1:1. This may be related to the disruption of both covalent and non-covalent bonds, which leads to folding of the protein structure and consequently exposes more internally active groups [49]. Therefore, it can be concluded that the addition of a small amount of the A-subunit can enhance the mechanical properties of SLP gels and that ultrasonic significantly improves the mechanical properties of A-SLP gels.

Before ultrasonic, at varying ratios of B-subunit to SLP, only elasticity exhibited an upward trajectory with increasing B content. Furthermore, following ultrasonic at varying ratios, the trend remained consistent with the previous results. However, the observed significance was not as pronounced as that observed before ultrasonic. Therefore, it can be concluded that the addition of the B-subunit enhances the elasticity of SLP gels. Disruption of other mechanical properties may be due to the distribution of low-molecular-weight B-subunit aggregates in the crosslinked structure of SLP, which hinders the formation of a continuous, homogeneous network.

#### 3.2.3 Rheological properties

**Fig. 6:**
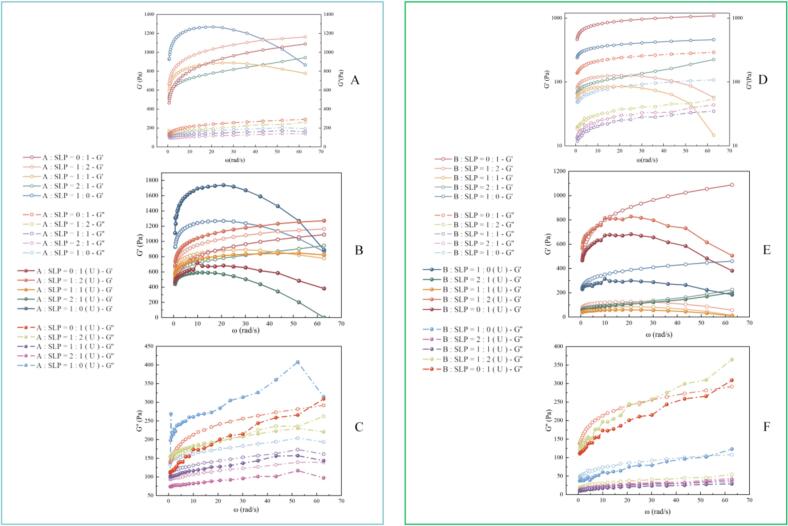
Storage or elastic modulus (G′) and loss or viscous modulus (G″) of mixed gel with different ratios of A, B, and SLP. (Figure A depicts the G′ and G″ of A-SLP before ultrasound. Figure B illustrates the G″ of A-SLP both before and after ultrasound. Figure C presents the G″ of A-SLP before and after ultrasound. Figure D illustrates the G″ and G″ of B-SLP before ultrasound. Figure E depicts the G″ of B-SLP before and after ultrasound, and Figure F presents the G″ of B-SLP before and after ultrasound).

As illustrated in Fig. 6A-F, the storage modulus (G') of the gels was greater than the loss modulus (G’), with no crossover phenomenon. The findings indicate that the gels exhibit typical viscoelastic behavior [50], [51]. Furthermore, the G' and G″ curves of all samples exhibited a frequency dependence, which was attributed to the higher energy stored by cross-linking between proteins [52]. These results demonstrate that subunit A forms a stronger elastic gel network than subunit B, consistent with the findings of Ju et al. [53]. The gels with an A:SLP ratio of 1:2 exhibited the highest G' and G″ values when mixed at different ratios throughout the frequency range. This may be attributed to the enhanced electrostatic repulsion of the crosslinks between the protein molecules. For A:SLP = 1:1, B:SLP = 1:1, and B:SLP = 1:2, the G' values of the gels decreased with increasing frequency, indicating the pronounced dependence on frequency of the three gel samples. In contrast, the slopes of the G' versus frequency plots at the other ratios remained unchanged, suggesting that the frequency dependence of the G' has a minimal effect [50].

Following ultrasonic, the G' of the gels at A:SLP = 1:2 and B:SLP = 1:2 increased, with a notable enhancement in the elasticity of the protein gels in comparison to the other samples. This may be attributed to the fact that ultrasonic disperses larger protein particles, especially SLP, increasing solubility and surface area, which further promotes collisions and tighter aggregation of proteins in solution by activating hydrophobic and thiol groups [54], [55]. Additionally, Zhang et al. [25] proposed that substantial alterations in the secondary structure have a notable impact on the elasticity of gels. When considered along with the secondary structure outcomes, the A:SLP gels and B:SLP = 2:1 gels exhibited lower G′ values both before and after ultrasonic.

**Fig. 7:**
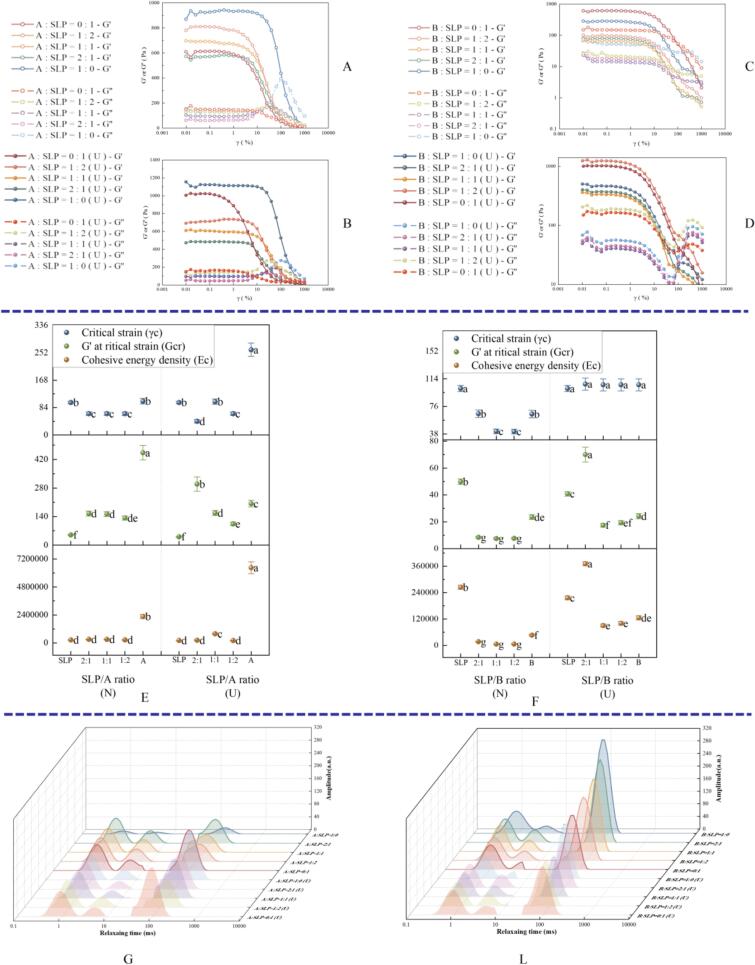
Large amplitude oscillatory shear behavior and critical strain (γ_c_), G' at critical strain (G_cr_) (A-D), cohesive energy density (E_c_) (E-F), and LF-NMR T2 relaxation curve G-L) of mixed gel with different ratios of A, B, and SLP.

Strain scans of the protein gels are shown in Fig. 7A-D. The flat region of the strain scan curve falls within the LVE range of the sample, when the values of G' and G' are essentially stable [56]. Upon exceeding the critical strain, a notable decline in the values of G' and G' is observed, indicative of a nonlinear LVE state [57]. The addition of A and B subunits resulted in a reduction in G' and narrowing of the LVR range, which is deleterious to the network structural elasticity and stability of the SLP-based system. However, ultrasonic increased the LVR range for A:SLP = 1:1 and B:SLP = 1:2 gels. Ultrasonic gel samples exhibited a wider LVR range and greater resistance to external damage, primarily because of their enhanced ordered structure [58].

To provide further comparison of the gel hardness of A-SLP and B-SLP gels before and after ultrasonic, the critical strain (γ_c_), G′ (G_cr_) at the critical strain, and cohesive energy density (E_c_) were calculated. The results are shown in Fig. 7E and F. Following ultrasonic, an increase in γc was observed, indicating a longer LVR and enhanced viscoelasticity of the gel under larger deformations. Additionally, an increase in Gcr was noted when the A:SLP ratio was 1:1, and an increase in Ec was observed at a ratio of 1:2, further indicating that the strength of the gel structure improved after ultrasonic [59]. However, there was no significant increase in the Gcr and Ec values of the B-SLP gels after ultrasonic, indicating that the process may have enhanced protein–protein or protein–water interactions, enabling the gels to withstand greater deformation.

#### 3.2.4 Low-field NMR

The transverse relaxation time (T2) of low-field NMR can be used to characterize the binding state of the gel system and water, as well as the mode of interaction of the solute molecules. The four T2 peaks of T2b (0.25–1.51 ms), T21 (2.31–7.05 ms), T22 (8.48–151.99 ms) and T23 (351.12–1629.75 ms) correspond to (1) bound water (2) immobilized water (3) free water (4) extra water outside the gel system, respectively [60].

As illustrated in Fig. 7G, the T22 relaxation time increased with the incorporation of the A-subunit into A-SLP gels, whereas there was no notable alteration in T2b and T21. These findings suggest that the introduction of the A-subunit diminished the strength of the hydrophobic interactions, facilitated the mobility of water molecules, and consequently compromised gel stability. Following ultrasonic, T22 exhibited a reduction in the relaxation time, a trend that was more pronounced in gels comprising the majority of subunit A. The findings suggest that addition of the A-subunit resulted in an increase in the fixed water content after ultrasonic of gels.

As illustrated in Fig. 7L, in B-SLP gels, the relaxation peak shifted towards lower relaxation times and the peak increased with the increase in B-subunit content, indicating that the addition of B-subunits enhanced cross-linking between protein molecules, leading to an increase in the amount of fixed water content [61]. The reduction in water molecule mobility and reinforcement of gel stability were ascribed to the considerable intensification of hydrophobic interactions. Conversely, following ultrasonic, addition of the B-subunit resulted in a diminished shift of T22 towards a lower relaxation time, enhanced molecular mobility, and compromised gel stability. This can be attributed to the fact that the particle size of the B-subunit was insufficient for optimal gel formation.

In conclusion, the addition of the A-subunit increased the mobility of water molecules in SLP gels, but the formation of a continuous and homogeneous structure after ultrasonic increased the immobilized water content, whereas the addition of the B-subunit decreased the mobility of water molecules, but the mobility of water molecules was increased after ultrasonic.

### 3.3 Principal component and correlation analyses

**Fig. 8:**
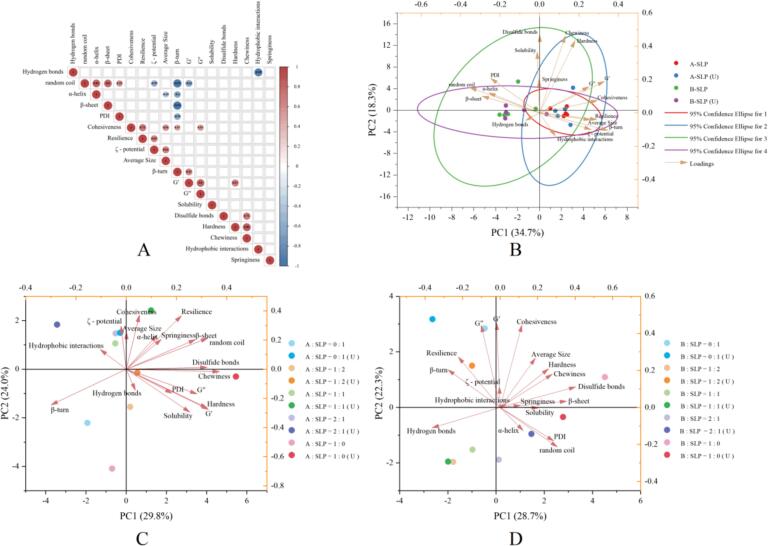
Pearson correlations (* P < 0.05) (A) and PCA biplot (B-D) of physicochemical properties of proteins and properties of gels.

Fig. 8A illustrates the correlation between the physicochemical properties of the proteins and the rheological and textural parameters of the gels as determined by the Pearson correlation coefficient (r). The r varied between −1 and 1 and was color-coded to demonstrate the strength of the correlation. In four groups of samples, a positive correlation was observed between cohesiveness and average size (r = 0.56), β-turn (r = 0.47), and resilience and ζ-potential (r = 0.65). Additionally, a positive correlation was noted between G' and β-turn (r = 0.63) and random coil (r = 0.62). Furthermore, a positive correlation was identified between chewiness and disulfide bonds (r = 0.71). Furthermore, the average size was positively correlated with the ζ-potential (0.63) and negatively correlated with α-helix (0.45).

The findings support the conclusion that following the unfolding of the denatured structure through heating, a greater exposure of the charged groups of the protein and larger particle size increase the cohesiveness and resilience of the gel. Furthermore, the higher content of β-turn after unfolding/refolding of the protein also improves the cohesiveness and G' of the gel. The weak correlation between the remaining properties may be attributed to the intricate nature of the factors influencing the gel properties, which encompass interactions between protein molecules, gel chain thickness, and the degree of homogeneity or roughness in the gel structure [39].

As illustrated in Fig. 8B, the distribution of each group of samples before and after ultrasonic varied in different dimensions (elliptical). Notably, the ellipse angles of A-SLP and B-SLP(U) were identical, whereas the distribution range of B-SLP(U) was larger than that of A-SLP. The finds are consistent with the observation unfavorable tendency towards gel formation of the B:SLP = 1:1 gel after ultrasonic. This may be due to the smaller size of the protein particles. However, the gel formed at the B:SLP ratio of 1:2 after ultrasonic exhibited superior gel strength. As illustrated in Fig. 8C and D, ultrasonic enhanced the properties of the A-SLP gel, despite the initial dispersion of the samples across distinct quadrants. However, the textural attributes of the gels improved only at B:SLP = 2:1 and 1:0.

### 3.4 Proposed mechanism of A, B and SLP gelation at the subunit level

**Fig. 9:**
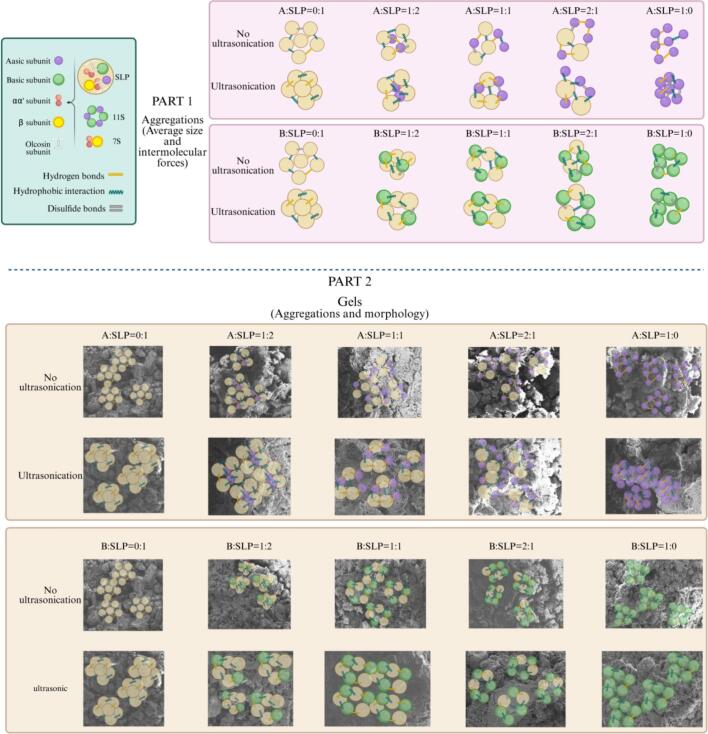
Schematic representation of the mechanism on gelation process of A, B, and SLP at subunit level.

Fig. 9 illustrates the mechanism of the formation of insoluble aggregates in the absence and presence of ultrasonic. The proportion of A-SLP resulted in larger particle than B-SLP. Additionally, the B-subunit exhibited a higher disulfide bonding content, which provided structural support. However, this content decreased following the mixing of SLP. This is presumably due to the lower solubility and unfavorable distance between the particles for disulfide bonding. In contrast, A-SLP displayed higher solubility, and the soluble proteins in the gel formation process filled in between the particles, with this filling effect being more obvious in SEM images at 1000× magnification.

Ultrasonic accelerated protein structural decomposition and increased the concentration of soluble proteins. Although the protein particle size decreased, ultrasonic caused the protein to expand in volume. Consequently, the impact of ultrasonic on the gel can be summarized as follows. Following ultrasonic, protein swelling results in a particle size that did not undergo significant reduction. This further results in a gel strength consistent with that of the pre-sonicated gel, as well as an increase in soluble proteins within the gel network, which further improves gel elasticity. However, it is important to note that the specific improvement in gel strength also requires attention to changes in protein structure, including secondary, tertiary, and intermolecular forces.

## 4 Conclusion

In this study, soy protein gels with varying A:SLP and B:SLP ratios were prepared using thermally induced medium-power ultrasonic (400 W) pre-treatment. Before ultrasonic, the addition of A-subunit increased the content of α-helix in the SLP gels and enhanced the rigidity of the gels; the addition of B-subunit increased the content of β-sheet structures and decreased the content of β-turn structures, which resulted in the formation of a more ordered and stable structure. After ultrasonic, the addition of A-subunit decreased the solubility of proteins in SLP gels, the particle size became smaller, the energy storage modulus and yield stress increased, the elasticity of the gel and the homogeneity of the network were improved, and the microscopic improvement increased the water content in the interstitial space of A-SLP gel matrix; the addition of B-subunit reduced the hydrophobic thermal aggregation, and at the same time, the disulphide bonding decreased, the particle size decreased, which was unfavourable for the formation of the gel matrix, and it also enhanced the mobility of water in the B-SLP gels and weakened the stability of the gels. This study elucidates the effect of ultrasonic treatment on the formation of soybean globulin-SLP hybrid gels at the subunit level, providing a theoretical basis for the synergistic utilization of soybean proteins between different components. ultrasonic has been shown to have an outstanding ability to dissolve large protein aggregates and swell globular protein structures. The role of ultrasonic technology in improving the physicochemical and functional properties of soy proteins should not be overlooked. Further efforts should be made to optimize the operating parameters involved in the ultrasonic process to maximize the gel properties of soy protein.

## CRediT authorship contribution statement

**Yuyang Huang:** Visualization, Software. **Jiyuan Liu:** Writing – original draft, Formal analysis. **Yongping Li:** Software. **Yongsheng Zhu:** Investigation, Data curation. **Gang Chen:** Validation. **Baoning Zheng:** Writing – review & editing. **Yixin Zhang:** Investigation. **Yang Li:** Resources, Project administration. **Xiuqing Zhu:** Writing – review & editing, Supervision.

## Declaration of competing interest

The authors declare that they have no known competing financial interests or personal relationships that could have appeared to influence the work reported in this paper.
